# Supplementary figures and images for: Molecular and clinicopathological implications of PRAME expression in adult glioma
Source: PLoS One. 2023 Oct 5;18(10):e0290542. doi: 10.1371/journal.pone.0290542 (PMC10553321; doi:10.1371/journal.pone.0290542)

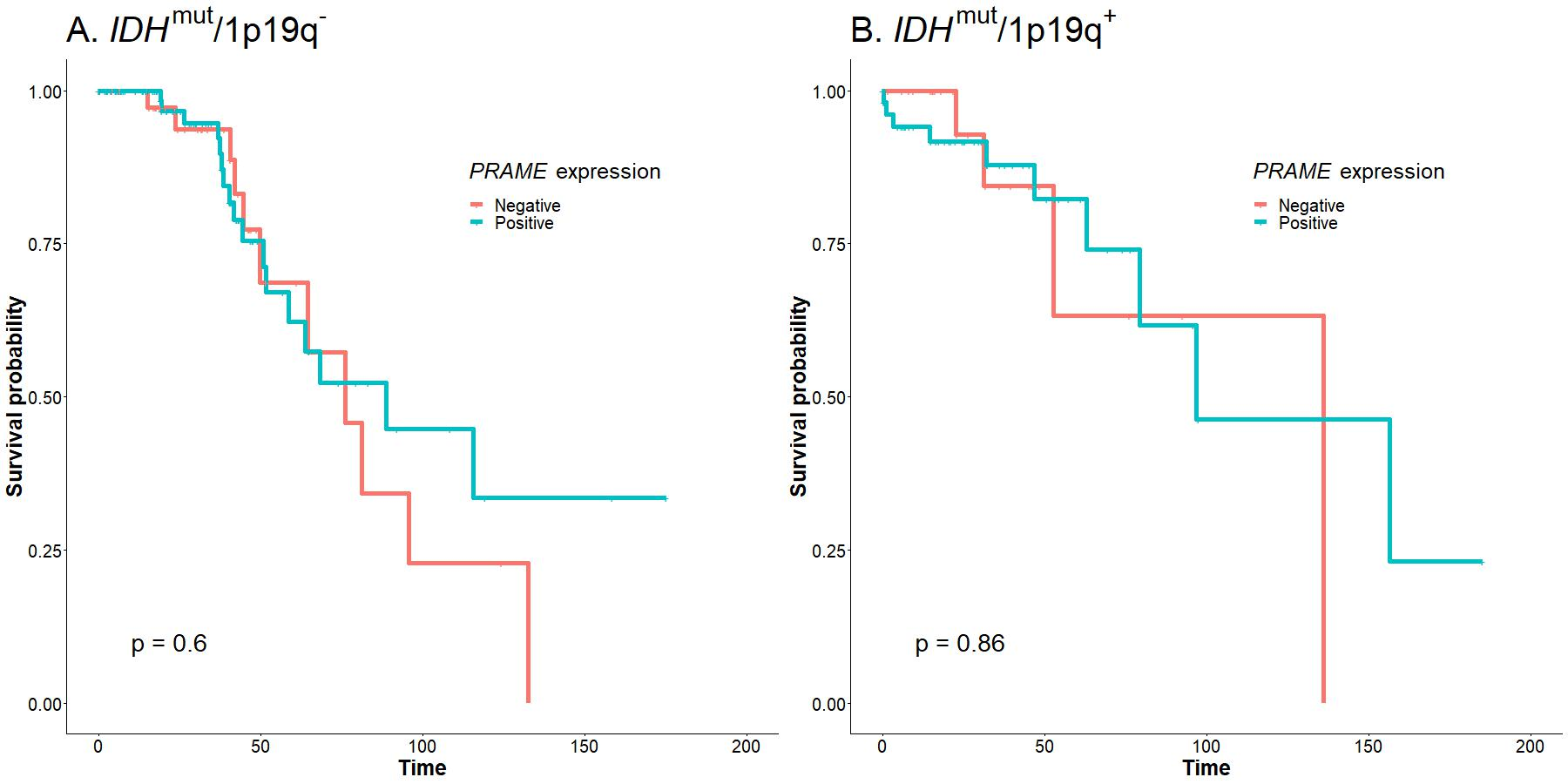

Supplement: S1 Fig — Kaplan-Meier curves compare the survivorship of PRAME-positive and PRAME-negative tumors in IDH-mutant gliomas with (A) and without (B) 1p/19q co-deletion. (TIF) [file pone.0290542.s001.tif]
