# Supplementary material for: Molecular and clinicopathological implications of PRAME expression in adult glioma
Source: PLoS One. 2023 Oct 5;18(10):e0290542. doi: 10.1371/journal.pone.0290542 (PMC10553321; doi:10.1371/journal.pone.0290542)
Supplement: Attachment — Submitted filename: Response_R1.docx [file pone.0290542.s003.docx]

**To the editors:**

Thank you for processing our manuscript. In this revised version, we made modifications to the author board and analyses. Although there many alterations added, there are 3 main changes.

First, we regret to inform you that we removed our co-author, Kathryn Eschbacher, from the author board. It should be noted that there was no conflict of interest in this project and this removal was made solely based on the personal reasons of this author.

Secondly, we re-run the analyses by using our published preprocessing pipeline [PMID: 37253389] and additionally used data cbioportal for a higher quality of data. The results were similar but the data presentation can be different due to different random seeds of analysis. All the results related to Figures and Tables were revised accordingly.

Thirdly, we modified **Table 2**, focusing on 4 distinct multivariate analyses of *PRAME* status adjusted for clinical characteristics, genetic mutations (WGS-available data), *TERT* promoter mutations (WES-available data), and copy number alterations (7+/10-, EGFR amplification, and *CDKN2A/B* homozygous deletion). The reason for these modifications is due to the heterogeneity of data availability of different data types (clinical information, WGS, WES, and copy number array). Stratified survival analysis of *PRAME* status by new classifications was done independently by Kaplan-Meier analyses.

**Reviewer #1: In this manuscript, Khang Le et al. explore the publicly available human cancer atlas to reveal the role of PRAME gene expression and its biological and clinicopathological significance in gliomas and established it as a potential prognostic biomarker for glioma. The study has enormous translational importance, as they justified the prognostic signature in potential study cohorts for further clinical applications.**
Thank you for reviewing our manuscript.

**The authors should address the following issues to consider this manuscript, primarily standard. However, the description is sometimes concise, and the authors should provide more details.**

**Minor comments:**

**1. The general description of pertaining to glioma vs glioblastoma should be mentioned in introduction section of the manuscript.**

We added a few sentences describing the context of WHO classification regarding glioma and glioblastoma in Introduction as follows:

“Adult gliomas are a heterogeneous and common group of brain cancers with unclear cell-of-origin [5]. The biological profile of gliomas has been studied with respect to histology, epigenetic, genetic characteristics, cell-of-origin, and tumor microenvironment [6,7]. There are highly diverse oncogenic mechanisms contributing to gliomagenesis and tumor progression, including Wnt/𝛽-catenin [8], PI3K/Akt/mTOR [9], TGF-𝛽 [10], and mesenchymal transition, among many others [11]. Important genetic abnormalities affecting the prognosis of glioma patients consist of *IDH1/2* mutations, *CDKN2A/B* homozygous deletion, *EGFR* amplification, *TP53* mutations, *ATRX* mutations, *TERT* promoter mutations, and 7 gain 10 loss chromosomal abnormalities [12]. The recent World Health Organization (WHO) classification of Tumours of the Central Nervous System (CNS) emphasizes that glioma can be divided by *IDH* mutation and 1p/19q codeletion status. *IDH*-wildtype astrocytoma has more advanced clinicopathological progression and tumor with grade 4 is referred to as the “glioblastoma” category. Glioblastoma is diagnosed by the absence of *IDH* mutation and one of the high-grade features, including high-grade morphology, *TERT* promoter mutation, 7 gain/10 loss chromosomal abnormality, or *EGFR* amplification. However, the prognostic factors of glioma are still under investigation.”

**2. The author should discuss the role of IDH1-mutation in the prognosis of glioma.**

Thank you for your comments.

We added more details about *IDH* mutation in Introduction to clarify the background in the previous comment.

**3. Should make a diagram of signaling pathways that are upregulated and downregulated
in PRAME-positive gliomas and PRAME-negative gliomas.**

We added **Figure 1D** that describes pathways that are down-regulated or up-regulated regarding the *PRAME* expression status.

**Reviewer #2: In this manuscript, the authors investigated and claimed that PRAME positivity is an independent prognostic factor for poor outcomes. They suggested that its over-expression can indicate different biological and clinicopathological profiles, such as age, grade, and EGFR amplification, in adult glioma. High PRAME positivity was found to be solely associated with wtIDH glioma, glioma DNA methylation landscape, and poor outcome. While PRAME is an important molecular and clinicopathological marker for different cancers including adult glioma, the reviewer believes that the present work lacks novelty as similar conclusions have already been established in previous publications. Furthermore, the present work lacks a mechanistic approach, and several aspects of the manuscript still need to be properly addressed."**

Thank you for reviewing our manuscript.

**Major concerns:**

**1. It has already been established in previous publications that PRAME is expressed in adult glioma and is associated with poor prognosis. [Wei et al., J. Neurooncol. 2019 Apr; 142(2):375-384. doi: 10.1007/s11060-019-03110-5.] [Zhang et al., J. Neurooncol. 2008 May; 88(1): 65-76. doi: 10.1007/s11060-008-9534-4.] [Zhao et al., Ann Clin Lab Sci. 2022 Mar; 52(2): 185-195.] The present study primarily utilized public databases, and no immunohistochemical data were provided to support their claim. Please provide reasons to support the uniqueness and novelty of this article compared to previous publications.**

Thank you for bringing these studies to our attention. We believe that the purpose of the aforementioned studies is different from our present work. We focused on the biological and clinicopathological investigation of *PRAME* expression in a more comprehensive way, including epigenetic, genetic, and gene expression.

We added a paragraph to discuss this issue as follows:

“The significance of *PRAME* expression has been mentioned previously. Wu et al [20] developed a *PRAME*-containing formula for risk score, which was inferred from the regression model. This score quantified the risk of Karnofsky performance score, but not the prognosis itself. Therefore, the inference of *PRAME* prognostic value is plausible but weak. The other study by Zhang et al [21] mainly compared the *PRAME* expression between different types of brain tumors, including subtypes of astrocytic and non-astrocytic tumors. However, it can be difficult to conclude that *PRAME* expression is an independent prognostic factor by the current evidence. On the other hand, the goal of our study is to focus on the biological and clinicopathological characteristics of *PRAME*. Multiple analyses were performed to provide more concrete proof of the significance of this gene in glioma.”

**2. According to the WHO 2021 guidelines, wtIDH gliomas are clustered together in grade 4, including diffuse astrocytoma, anaplastic astrocytoma, glioblastoma, and glioblastoma NOS.**

We believe that Glioblastoma, *IDH*-wildtype is defined when there is one or more of the following features: microvascular proliferation, necrosis, *TERT* promoter mutation, *EGFR* gene amplification, and 7+/10- chromosome copy-number changes according to WHO classification 5^th^ edition. Therefore, astrocytic tumors with *IDH* wildtype status and no other feature are ill-defined and should be classified as Astrocytoma, NOS. Therefore, we revised the classification in a more detailed way, following our recently published paper [PMID: 37253389].

We added details about our revised data processing pipeline as follows:

“**Data processing**

The Human Cancer Atlas (TCGA) database consists of many datasets. We extracted cases from the TCGA-GBM and TCGA-LGG projects. Only cases with available gene expression profiles (GEP) and primary tumors (no recurrent or metastatic tumors) were included in the study. To adapt to the new WHO classification, cases with grades 2/3 in the previous studies [13,14] were reclassified into grade 4 as follows: (1) the presence of both *IDH1/2* mutation and *CDKN2A/B* homozygous deletion, or (2) the absence of *IDH1/2* mutations and the presence of at least one of the following abnormalities: *TERT* promoter mutation, *EGFR* amplification, and 7 gain 10 loss chromosomal abnormality. Other histopathological grading features such as microvascular proliferation and pseudopalisading necrosis were assumed to be included in previously evaluated grade IV gliomas of the original studies [13,14]. Tumors with the absence of *IDH1/2* mutations and no other high-grade morphological and genetic features mentioned above were reclassified as Astrocytoma, Not Otherwise Specified (NOS). Tumors with the presence of both *IDH1/2* mutations and 1p/19q codeletion were categorized as Oligodendroglioma. Mixed glioma was re-distributed into new categories based on *IDH1/2* mutation and 1p/19q codeletion status. This reclassification was published in our previous paper [15]. The difference in data processing between this study and our previous one was that we used cbioportal for cancer genomics (<https://www.cbioportal.org>) datasets that are related to TCGA-GBM and TCGA-LGG projects, including (1) Brain Lower Grade Glioma (TCGA, Firehose Legacy), (2) Glioblastoma Multiforme (TCGA, Firehose Legacy), and (3) Merged Cohort of LGG and GBM (TCGA, Cell 201**6**). This difference led to a slight inconsistency in the total number of glioma patients.”

**The present study claims a positive correlation between PRAME and wtIDH glioma, but it doesn't clearly explain the correlation between PRAME and these wtIDH gliomas. It is important to mention the expression status of PRAME in these specific groups of gliomas.**

We added a boxplot (**Figure 1E**) to illustrate the *PRAME* expression status in each revised category of glioma.

3**. PRAME is highly immunogenic and serves as a robust target for immunotherapy. Certain drugs, such as Decitabine, can increase PRAME expression to enhance T-cell-mediated toxicity against GBM, which contradicts the findings of the present study. [Ma et al., Neuro-oncology. 2022 Dec; 24(12), 2093-2106]. The present study lacks an exploration of the immunological aspects of PRAME in gliomas. It is important to investigate the correlation between different immunological populations, pro-inflammatory signaling cascades, and PRAME.**

Thank you for your information.

We added a section of analysis named “**Analysis of the association between tumor microenvironment (TME) and *PRAME* expression**” as follows:

“**Analysis of the association between tumor microenvironment (TME) and *PRAME* expression**

Given that *PRAME* is associated with cytotoxic T-cell activation and killing in glioblastoma [15], we compared the immunologic cell population between *PRAME*-negative and *PRAME­*-positive gliomas. GSVA of cell type-specific gene sets [16] was performed to calculate the activity (ES) of 17 cell types, including B cells, T cells, T helper, Th1, Th2, TFH, Th17, Treg, CD8 T cells, T gamma delta, cytotoxic cells, NK cells, dendritic cells, eosinophils, macrophages, mast cells, and neutrophils (**Figure 5**). There were increased activities of T cells (p<0.001), Th2 (p<0.001), Th17 (p<0.001), cytotoxic cells (p=0.028), macrophages (p<0.001), and neutrophils (p<0.001) in *PRAME*-positive gliomas while there was reduced activities of TFH (p<0.001) and CD8 T cell (p<0.001).”

We also added a corresponding discussion paragraph:

“*PRAME* can also be associated with glioma TME. A previous study showed that Decitabine can increase *PRAME* expression and, thus, enhance the T-cell-mediated cytotoxicity, which makes *PRAME* an interesting target for immunotherapy [15]. However, TME of cell line can be difficult to interpret because the stromal or microenvironment context of cancer in vivo is different from that of cell line condition. Our study showed that *PRAME* higher expression was related to increased cytotoxic cell, macrophage, and neutrophil activity but it was also associated with many immune modulating cells such as Th2, Th17, and TFH. Therefore, *PRAME* expression is in a complicated relationship with many immunologic cell populations, not only cytotoxic T cells.”

4. **Among 665 patients, 170 patients were PRAME-negative, and 495 patients were PRAME-positive. Moreover, within the PRAME-positive population, 481 had low PRAME expression, and only 14 had high PRAME expression. The study compared the PRAME-positive and -negative populations for various biological and clinicopathological parameters. However, the study didn't explain the biological and clinicopathological differences between the low and high PRAME-expressing populations and their relationship with patient survival. It would be worthwhile to compare the biological parameters between the 481 low and 14 high PRAME-expressing sample sets. Additionally, the number of samples in the high PRAME-expressing set is too small to draw conclusions. Increasing the sample size for the high PRAME-positive population is advisable.**

Thank you for your suggestion. However, it is quite difficult to define a cutoff of *PRAME* overexpression. This is emperically determined by the data at hand and defined by >1, >3, or >5 FPKM. For example, a previous study used FPKM>1 to define higher expression [PMID: 27577089]. Therefore, we believe that it is better to follow these recommendations. In our data, a vast number of cases had *PRAME* read count<1. Therefore, >1 FPKM is an appropriate cutoff for determining high expression or overexpression.

As the reviewer mentioned, the sample size of *PRAME*-overexpressing cohort was small to create reliable results. Therefore, to avoid multiple hypothesis problem, we only performed survival analysis for this cohort. We additionally applied Benjamini-Hochberg correction for the pairwise comparison in this survival analysis to solidify the result. The analysis of other biological parameters, which requires a lot of hypothesis testing, can be performed but it can lead to false positive results and misleading conclusions. Therefore, we believe that it is better not to over-do the investigation of the *PRAME*-overexpressing cohort.

5. **In the Results section, the authors mentioned that PRAME-positive tumors expressed higher activity in the PI3K-AKT-mTOR, P53, and IL2-STAT5 pathways in gliomas. However, Fig 1C doesn't correlate with this statement, as it shows upregulation of KRAS, TGFb, and Notch signaling pathways in PRAME-positive tumors. Additionally, in Fig 3A, 3B, and 3C, it seems that the PRAME-positive population has longer survival than the PRAME-negative population, although statistical significance is lacking possibly due to the small sample size. Similarly, in Fig 4 and Supplementary Fig A & B, the PRAME low-expressing population tends to have longer survival than the PRAME-negative population. In Fig 3D, PRAME-positive mutant IDH1/2 shows higher survival than PRAME-negative wt-IDH1/2 population. Please provide a proper explanation of the data.**

Thank you for pointing out this observation. In Figure 3A, *PRAME*-positive and *PRAME*-negative glioma patients had difference in survival patterns (p<0.001). We concur with you that the *PRAME*-negative cohort survived better in the >10 years of follow-up. Unfortunately, we do not have a clear explanation for this observation. Although it seems that *PRAME*-negative cohort deceased sooner than *PRAME*-positive cohort in the period of >10 years of follow-up, there may be many confounding factors because of the long follow-up time. Another confounding factor is sample size. The size of *PRAME*-positive cohort was much larger than *PRAME*-negative one. Therefore, the positive cohort was likely to have more outliers than the negative cohort. Generally, first years of follow-up are more reliable prognostic indicator compared to longer term of follow-up. To avoid this confusing phenomenon, we limited the x-axis from 0 to 10 years of follow-up, which eliminates such observation of the outliers.

6. **It has already been established that PRAME overexpression can inhibit the growth of breast cancer and leukemia. However, PRAME overexpression in glioma is associated with poor outcomes. The present study compared the hallmarks of apoptosis between PRAME-positive and -negative adult glioma but found no differences in apoptotic hallmarks between the two groups. Please explain these findings in detail with underlying mechanisms. Also, the specific role of PRAME in retinoic acid signaling in glioma malignancy is unknown. Since the present study lacks a mechanistic approach, it is important to consider the correlation of PRAME expression with retinoic acid signaling status in wtIDH gliomas.**

Thank you for your suggestion. We experimentally run pathway analysis to investigate the activity of retinoic acid signaling (the full gene set of this pathway is in the link below). The enrichment score (ES) of *PRAME*-negative and *PRAME*-positive glioma were compared by two-sample independent t-test, which was similar to other pathways. There was no significant difference between *PRAME*-negative and *PRAME*-positive samples (p=0.446**)**. Therefore, the role of *PRAME* in glioma may not be related to its suppression of retinoic acid signaling. However, we believe that it is better not to include this analysis into the manuscript because this analysis may not be relevant to the main stream of analysis and it can also cause confusion to readers.

The link to retinoic pathway:

<https://www.gsea-msigdb.org/gsea/msigdb/human/geneset/REACTOME_SIGNALING_BY_RETINOIC_ACID.html>

7. **Histone methylation or acetylation status, along with other epigenetic modifications, are important hallmarks of glioma development and progression. The present study did not establish any correlation between PRAME expression and histone methylation or acetylation status in glioma. Additionally, what is the correlation between G-CIMP and PRAME in glioma?**

Thank you. The lack of histone modification data is the limitation of the present study. We added a few sentences to the Discussion as follows:

“However, there were limitations in the present study. First, selection bias was a potential problem because this study used a public database. Second, our findings of *PRAME* positivity in *IDH*-mutant glioma were not significant potentially due to small samples, and, thus, sampling error. Therefore, a larger study of *PRAME* expression on *IDH*-mutant glioma can be helpful to examine the biological and clinicopathological relevance of *PRAME* positivity in these brain tumors. Third, protein expression data was not fully available and, therefore, cannot be analyzed. *PRAME* gene expression may be different from PRAME protein expression, which can be practically evaluated by immunohistochemistry. Hence, immunohistochemical studies are required to validate PRAME prognostic significance at the protein level. Additionally, data about histone modification is not available in TCGA-LGG and TCGA-GBM projects. Therefore, we were not able to analyze the relationship between *PRAME* expression and this epigenetic regulation. Finally, to our knowledge, there was no available information of chemotherapy and radiotherapy resistance in TCGA datasets. Hence, we were not able to investigate the relationship between *PRAME* expression and treatment response.”

In addition, we created **Figure 2E** to demonstrate the relationship between G-CIMP and *PRAME* expression in glioma.

To avoid confusion, we eliminate the analysis of t-SNE methylation clusters. We instead used the published methylation CIMP clusters and performed chi-square test. We modified the methylation Results section as follows:

“In the study cohort, there were a total of 476 tumors with available data about 450K DNA methylation. We performed t-SNE dimension reduction to explore the differences in the distribution of *PRAME*-negative and *PRAME*-positive glioma in DNA methylation hyperspace. This DNA methylation space can be interpreted as the reduced representation of the CpG methylation landscape. We also included the Glioma CpG Island Methylator Phenotype (G-CIMP), which was published in a previous paper [33941250]. In this study, 476 tumors in DNA methylation space can be relatively divided into two unsupervised tSNE clusters, small (right, lower corner) and large clusters (left and upper part) (**Figure 2A-D**). The small cluster densely consisted of *PRAME*-low and *PRAME­*-overexpressing samples while the larger cluster had a significant portion of *PRAME*-negative samples (**Figure 2A**). The revised subtype (**Figure 2B**), CIMP clusters (**Figure 2C**), and *IDH* status (**Figure 2D**) were strongly associated with these 2 clusters. **Figure 2E** shows a heatmap of distribution of *PRAME* expression status within CIMP clusters. There were significant difference in the distribution of *PRAME* expression status (chi-square test, p<0.001). This discrimination can be seen in LGm6-GBM (6/12 vs. 7/484 vs. 0/170), classic-like (1/12 vs. 63/484 vs. 4/170), and mesenchymal-like (2/12 vs. 87/484 vs. 9/170).”

8. **Radioresistance and chemoresistance are major obstacles in the successful treatment of adult glioma. Therefore, it would be advisable to investigate the correlation between PRAME expression and treatment resistance properties in adult gliomas.**

Thank you for your comment. However, there was no information about chemotherapy and radiotherapy resistance in the TCGA datasets. Therefore, we listed your comments as a limitation of our study.

**Minor concerns:**

1. **Please add the references at the end of the third sentence in the Introduction section.**

We added citations at the end of the third sentence in Introduction section.

2. **Please mention the figure/table numbers in the Results section at the end of the statements (a) "The association between PRAME expression and WHO grades was statistically significant" and (b) "Conversely, IDH1/2 mutations (87.9%) and ATRX mutations (39.8%) were significantly more common in PRAME-negative cases" under the sub-heading entitled "PRAME-positive gliomas had more advanced grades and adverse outcomes."**

Thank you. The referred paragraph was written as a description of Table 1 and it is mentioned in the first sentence of this paragraph:

“**Table 1** summarizes the clinicopathological characteristics of *PRAME*-negative and *PRAME*-positive gliomas.”

3. **Properly mention the title of the X-axis in Fig 3A-D.**

We changed the title of the x-axis from “Time” to “Follow-up Time (Years)”.

4. **On the title page, it should be ORCID, not ORCHID. Please correct this typo error.**

We corrected this error.

5. **Please thoroughly check the manuscript for grammatical and typographical errors.**

Thank you for your comments.

6. **Please rephrase the second sentence of the Discussion section, as it appears confusing to readers.**

We modified the sentence “In gene expression analysis, we explored *PRAME* read counts and found that *PRAME* expression was generally low in these brain tumors. A significant number of tumors showed no *PRAME* expression.” into “In gene expression analysis, there was a large number of gliomas showing no *PRAME* expression while a few numbers of tumors possessed high levels of *PRAME* expression. The remaining tumors generally showed low *PRAME* expression.”
